# Detecting the socio-economic drivers of confidence in government with eXplainable Artificial Intelligence

**DOI:** 10.1038/s41598-023-28020-5

**Published:** 2023-01-16

**Authors:** Loredana Bellantuono, Flaviana Palmisano, Nicola Amoroso, Alfonso Monaco, Vitorocco Peragine, Roberto Bellotti

**Affiliations:** 1 Dipartimento di Biomedicina Traslazionale e Neuroscienze (DiBraiN), Università degli Studi di Bari Aldo Moro, 70124 Bari, Italy; 2 Istituto Nazionale di Fisica Nucleare, Sezione di Bari, 70125 Bari, Italy; 3 Department of Economics and Law, Sapienza University of Rome, 00161 Roma, Italy; 4 Dipartimento di Farmacia-Scienze del Farmaco, Università degli Studi di Bari Aldo Moro, 70125 Bari, Italy; 5 Dipartimento Interateneo di Fisica, Università degli Studi di Bari Aldo Moro, 70126 Bari, Italy; 6 Dipartimento di Economia e Finanza, Università degli Studi di Bari Aldo Moro, 70124 Bari, Italy

**Keywords:** Scientific data, Applied physics, Computational science, Applied mathematics

## Abstract

The European Quality of Government Index (EQI) measures the perceived level of government quality by European Union citizens, combining surveys on corruption, impartiality and quality of provided services. It is, thus, an index based on individual subjective evaluations. Understanding the most relevant objective factors affecting the EQI outcomes is important for both evaluators and policy makers, especially in view of the fact that perception of government integrity contributes to determine the level of civic engagement. In our research, we employ methods of Artificial Intelligence and complex systems physics to measure the impact on the perceived government quality of multifaceted variables, describing territorial development and citizen well-being, from an economic, social and environmental viewpoint. Our study, focused on a set of regions in European Union at a subnational scale, leads to identifying the territorial and demographic drivers of citizens’ confidence in government institutions. In particular, we find that the 2021 EQI values are significantly related to two indicators: the first one is the difference between female and male labour participation rates, and the second one is a proxy of wealth and welfare such as the average number of rooms per inhabitant. This result corroborates the idea of a central role played by labour gender equity and housing policies in government confidence building. In particular, the relevance of the former indicator in EQI prediction results from a combination of positive conditions such as equal job opportunities, vital labour market, welfare and availability of income sources, while the role of the latter is possibly amplified by the lockdown policies related to the COVID-19 pandemics. The analysis is based on combining regression, to predict EQI from a set of publicly available indicators, with the eXplainable Artificial Intelligence approach, that quantifies the impact of each indicator on the prediction. Such a procedure does not require any ad-hoc hypotheses on the functional dependence of EQI on the indicators used to predict it. Finally, using network science methods concerning community detection, we investigate how the impact of relevant indicators on EQI prediction changes throughout European regions. Thus, the proposed approach enables to identify the objective factors at the basis of government quality perception by citizens in different territorial contexts, providing the methodological basis for the development of a quantitative tool for policy design.

## Introduction

The European Quality of Government Index (EQI), evaluated by the Quality of Government (QoG) Institute of University of Gothenburg^1^, measures the government quality perceived by European Union citizens. The index is computed starting from large surveys, in which citizens are asked about their perceptions and experiences of public sector corruption and impartiality, as well as good quality of public services. EQI aims to provide researchers and policymakers a guideline to understand how government approval varies both in space, within and across countries, and over time. The perception of government integrity is a matter of utmost importance, being one of the main factors in determining the level of civic engagement, which is identified by OECD as one of the life quality indicators.

In the last decades, concerns for the quality of government have come on top of the agendas of researchers and policymakers around the world. Improving institutional quality is, in fact, one of the Sustainable Development Goals (goal number 17) at the heart of the 2030 Agenda for Sustainable Development, adopted by all United Nations Member States in 2015^2^. In addition to promoting sustainable development, the quality of government is relevant for building confidence toward public institutions, which in turn strengthens the legitimacy and sustainability of political systems. This is especially important in difficult periods—including global shocks such as the 2007 financial crisis and the outbreak of the COVID-19 pandemic—during which low level of confidence in government may represent a barrier to the implementation of recovery procedures. In fact, high levels of confidence in institutions allow governments to act without resorting to coercion, thus reducing transaction costs and improving efficiency. The perceived quality of government is relevant from the perspective of public finance, as well. High levels of government quality suggest that citizens recognize greater compliance with the rules in the administrative action, discouraging informal sector activities. More in general, the higher the government quality within a country, the higher the incentives for citizens to behave honestly^3,4^. Last, improving the perception of government quality can be relevant to contain the wave of distrust in institutions, experienced in recent years by western societies.

**Figure 1:**
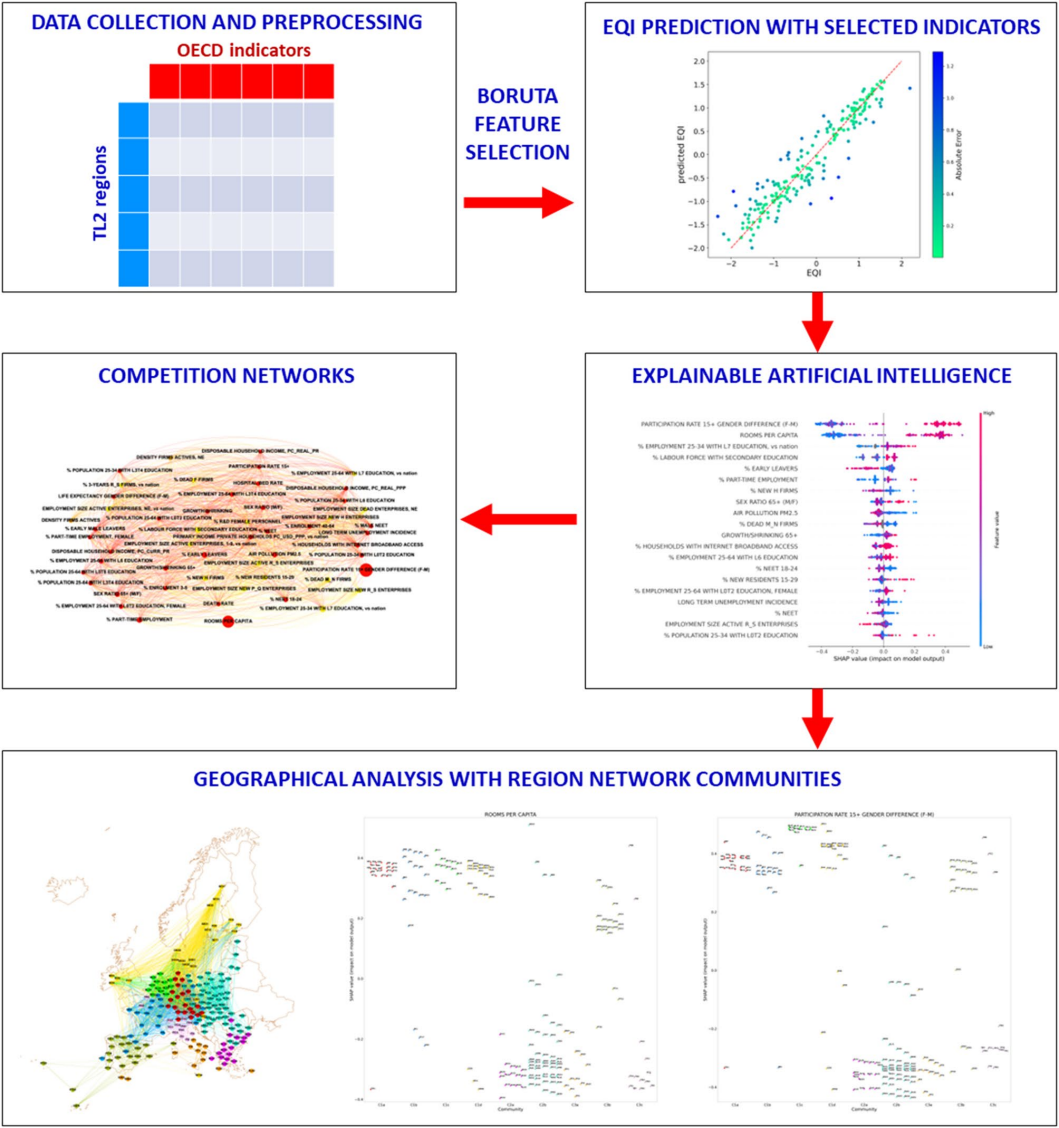
Scheme representing the workflow of the analysis, aimed at measuring the impact of territorial and societal development variables on the perceived government quality expressed by the European Quality of Government Index (EQI). The steps composing the analysis and the related findings are described in detail in the “Results” and “Materials and methods” sections.

In view of the aforementioned reasons, it is relevant for both evaluators and policy makers to understand possible hidden factors that affect the outcome of EQI for a given territory. The main purpose of our research is to investigate the relation between perception, at the basis of the EQI evaluation, and objective data, in diversified geographical and political contexts. In particular, we aim at measuring the impact of territorial and societal development variables on the perceived government quality expressed by the EQI, comparing across different regions the hierarchies of the most important indicators. A scheme of the workflow implemented in the present research is displayed in Fig. 1.

The analysis focuses on European Union regions at the subnational Territorial Level 2 (TL2), according to the OECD classification^5^. Our approach is based on characterizing each administrative region through a plurality of social, economic, cultural, environmental, and geopolitical factors. After data collection and preprocessing to discard redundant and irrelevant indicators^6^, we will move to the key step of our study, that consists in investigating the most influential features in determining the EQI, by evaluating their capability to predict the index value. Specifically, we will apply algorithms based on eXplainable Artificial Intelligence (XAI)^7^ to quantify the impact of indicators on the prediction of EQI made by an eXtreme Gradient Boosting (XGBoost) regressor. The XAI framework is a recently developed field of Artificial Intelligence (AI), which allows to increase the transparency and interpretability of machine learning models, meeting a crucial need, especially in their application to real-life cases^8–11^. Ideally, models should be based on a tradeoff between informativeness and uncertainty estimation on one hand, generalizability and transparency on the other^12,13^. Historically, the measure of informativeness, in terms of performance evaluation, and the uncertainty estimation, have been playing a major role^14–16^ so that, for many years, these represented the cornerstones of any AI model. Nowadays, an increasing attention is paid to generalization, namely the reliability of the model predictions on previously unseen data, and to transparency, namely the ability of the model to make the decision process as intelligible as possible^17–19^. Transparency becomes particularly crucial when dealing with ethical and juristic challenges related to the use of AI^20^. The compelling need for a unified view, combining informativeness, uncertainty estimation, generalization, and transparency, has led to the framework of techniques grouped under the common label of XAI. This approach, employed in our research, will enable to identify the factors that, in a variety of different socio-economic backgrounds, contribute to determine the perception of government quality by citizens, potentially providing the methodological basis for the development of a quantitative tool for policy design.

To provide a further insight in the multifaceted results of the XAI analysis and investigate the hierarchies of indicator importance, we will resort to the formalism of complex networks^21^, an instrument of complexity science that has become complementary to traditional statistical tools in many aspects of science and society, integrating conventional approaches and unveiling hidden information. Complex networks are increasingly used in a multidisciplinary scenario to investigate real-world systems, whose constituents are interconnected in non-trivial ways. The most prominent application fields of complex networks include economics^22–29^, human mobility^30^, social dynamics in critical events^31^, neuroscience^32–35^, genetics^36,37^, and even the development of policy design strategies, where they are used to encompass multifaceted context-based information in the evaluation of country^38^ and academic institution^39^ performance. Recent studies are also adding new instruments to the complex network toolbox, such as multilayer networks^40^ and network potentials^41,42^. In our case, we will construct competition networks^43,44^, in which indicators are nodes, and the strength of their connection is related with the tendency to switch their positions in the importance rankings. Features of outstanding relevance, which tend to stay on top of the rankings for most regions, will be characterized by weak connections in the competition network.

The last part of the study is devoted to a geographical characterization of our results. Also in this case, we will employ the complex network framework to provide a multidimensional representation of territorial development and citizen well-being, constructing a network in which regions represent nodes, and the strength of the connection between each pair of them increases with the similarity between indicator values. The tool of network assortativity^45^ will be used to explore possible relations between the strength of network connections, quantifying socio-economic similarity of two given regions, and the comparability of their EQI. Then, we will subdivide the set of European Union regions by deriving network communities^46,47^, which represent an optimized partition of the network determined by homogeneity in terms of the considered indicators. Communities will be employed to detect the relation between the importance of an indicator in EQI prediction and the development level of regions.

We will finally discuss the relevance of the results found throughout the analysis. In particular, we will interpret, also in the light of established knowledge and scientific outcomes, the emergence of particularly influential indicators in predicting EQI, and the effect of indicator variability among different regions of the same country.

## Results

The primary goal of this work is detecting, by using complex network and machine learning methods, the most impactful factors for the prediction of EQI, that quantifies the government quality perceived by European Union citizens for different TL2 regions. In this section, we describe the main findings of our analysis.

### EQI prediction and most influential features

The EQI 2021 index is available for 240 European Union regions, which can be traced back to 197 OECD subregions at Territorial Level 2 (TL2). However, the application of criteria related to the availability of OECD subregional indicators (see "Materials and methods") restricts our analysis to 195 TL2 regions. Such territorial indicators are collected from the OECD Regional Statistics database^5^, and can be classified in four groups: Business and Economy, Demography, Education and Labour, and Territorial and People Well-being. In particular, we choose to work with indicators that are available for at least two thirds of the considered TL2 regions, that are “intensive” (namely, not scaling with the region area or population), and that are not determined by subjective perception. Moreover, we add to the original dataset additional indicators, that we construct by comparing subregional data with the corresponding national values.

**Figure 2:**
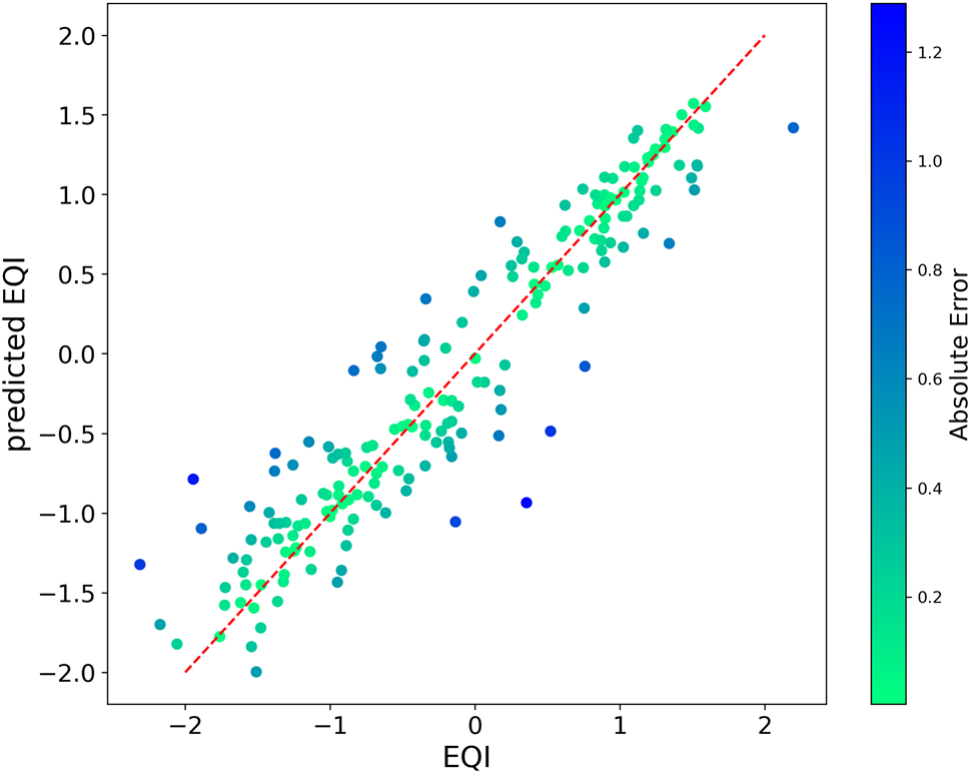
Scatter plot reporting the relation between the true value of EQI 2021 for European Union regions, and the prediction provided by XGBoost regression, operated in the leave-one-out mode. The regression algorithm performance is quantified by the value $r^2=0.8877$ of the coefficient of determination.

After undergoing a preprocessing workflow based on indicator availability and non-redundancy criteria, as outlined in detail in "Materials and methods", data are passed to a Boruta feature selection algorithm^6^. Supplementary Data S1–S4 reports a full list of the 612 indicators provided as input to Boruta. Out of them, such a feature selection process identifies 53 that are potentially relevant to predict EQI. Hence, we use the eXtreme Gradient Boosting (XGBoost) regression algorithm, trained in the leave-one-out mode on the 53 selected indicators, to predict the index value in the 195 TL2 regions. In the optimal parameter configuration ($\text {num}\_\text {parallel}\_\text {tree}=100$, $\text {max}\_\text {depth}=2$, $\text {n}\_\text {jobs}=100$, see "Materials and methods"), we obtain the results shown in Fig. 2. The effectiveness of regression is quantified by the following figures of merit,

1 $$\begin{aligned} r^2&= 0.8877 , \end{aligned}$$

2 $$\begin{aligned} P&= 0.9423 \quad \text {with } p<10^{-9}, \end{aligned}$$

3 $$\begin{aligned} MAE&= 0.2528 , \end{aligned}$$

4 $$\begin{aligned} RMSE&= 0.3424 , \end{aligned}$$

with $r^2$ the coefficient of determination, *P* the Pearson correlation between predicted and true index (with *p* the associated *p*-value), *MAE* the mean absolute error, and *RMSE* the root-mean-square error.

**Figure 3:**
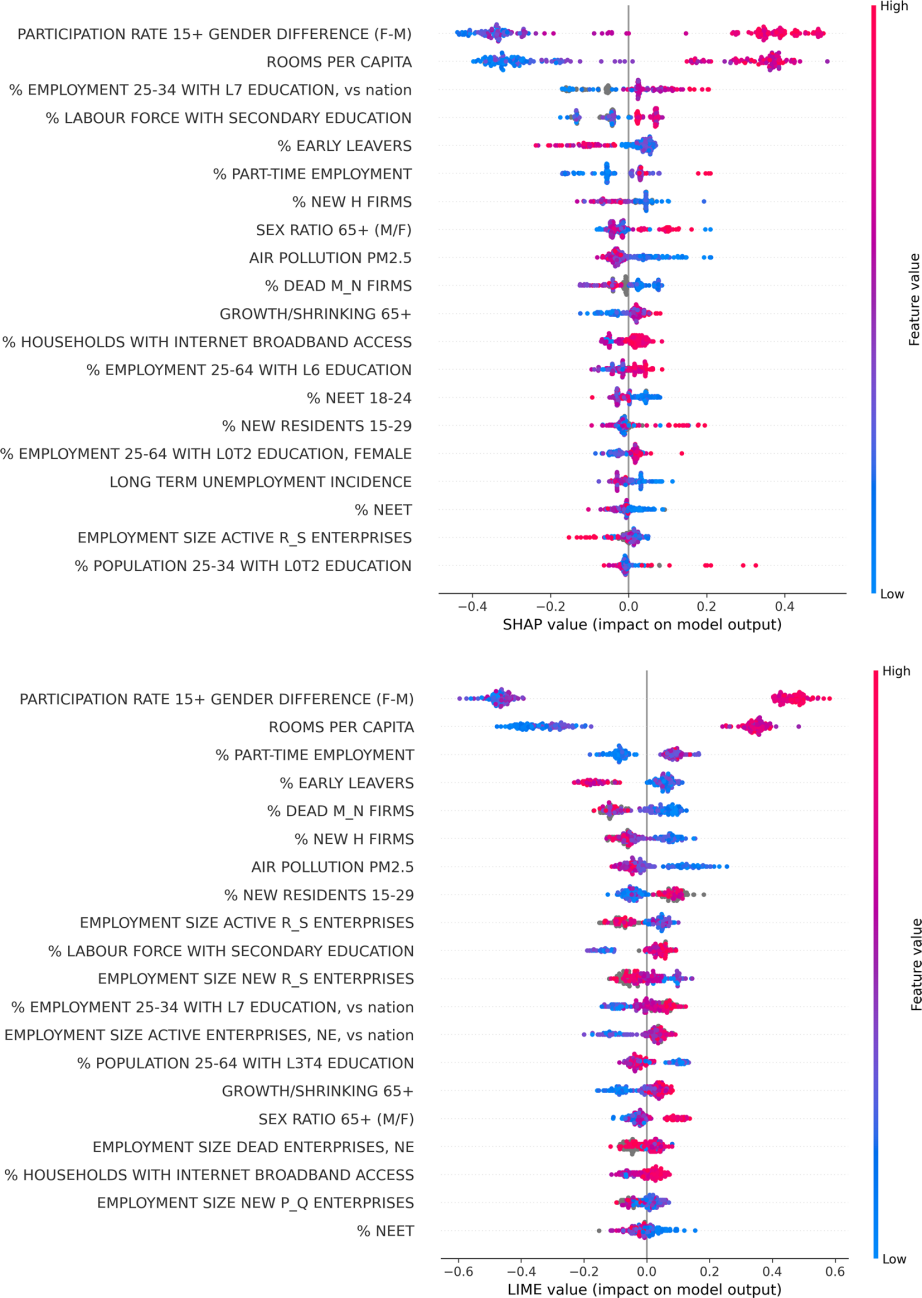
SHAP (upper panel) and LIME (lower panel) values corresponding to the most influential OECD indicators in the XGBoost prediction of EQI. In both plots, indicators are listed in descending order of mean absolute values of the corresponding SHAP and LIME, respectively. The definition of indicators is reported in the Supplementary Data S1–S4. The results highlight the large impact on EQI prediction of the difference between female and male labour participation rates for citizens over the age of 15 and the average number of rooms per inhabitant.

We then apply XAI algorithms to determine the most influential variables in the XGBoost regression. For each of the 195 leave-one-out cycles, we collect SHAP values and LIME values, which measure the impact of each indicator on the considered prediction. The results of the SHAP and LIME analyses are shown in Fig. 3, where each line reports a distribution of the values obtained in all cycles for a given variable. The plots, limited to the most influential indicators, highlight the large impact on prediction due to the features PARTICIPATION RATE 15+ GENDER DIFFERENCE (F-M), namely the difference between female and male labour participation rates for citizens over the age of 15, and ROOMS PER CAPITA, namely the average number of rooms per inhabitant. Notice that Supplementary Data S1–S4 contains an explanation of the concise indicator names reported in the text and in the figures.

### Competition network of indicators

Competition networks provide a tool to visualize and interpret the results of XAI analysis. Here, we construct two competition networks, starting, respectively, from the SHAP and LIME values associated to the 53 selected indicators for each region.

In a competition network nodes represent indicators, whose tendency to exchange their relative positions in a given set of rankings determines the weight of connections. This means that nodes that consistently place on top or bottom of the rankings tend to be characterized by the lowest connectivity (technical details are reported in "Materials and methods"). In our case, the two competition networks are based on 195 rankings determined, respectively, by the lists of absolute SHAP and LIME values associated to each given region. Notice that using the absolute values is necessary, since, as shown in the two panels of Fig. 3, both SHAP and LIME values, even for important features, tend to have vanishing average, since they can affect the outcome of regression either positively or negatively.

**Figure 4:**
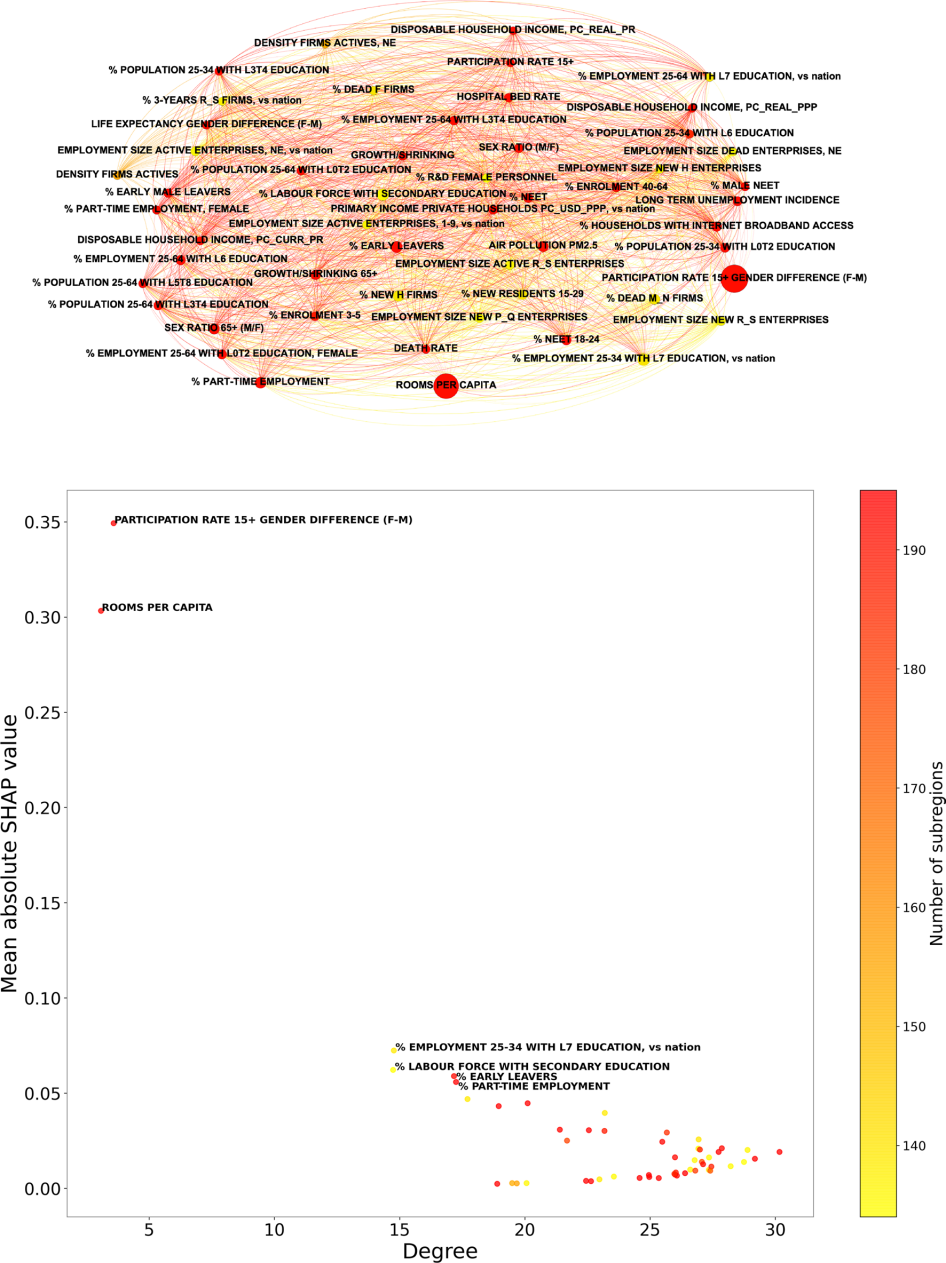
Top: competition network of the selected OECD indicators, determined from the importance rankings of their mean absolute SHAP values for each subregion considered in the study. Bottom: scatter plot illustrating the relation between the mean absolute SHAP value and the degree in the competition network, for indicator; labels are reported only for features whose mean absolute SHAP value exceeds 0.05. In both panels, the color of points indicates the number of regions for which the considered indicator is available in the dataset. In the top panel, the size of each node increases with the mean absolute SHAP value of the corresponding indicator. The results confirm that both PARTICIPATION RATE 15+ GENDER DIFFERENCE (F-M) and ROOMS PER CAPITA are outstandingly relevant in their capability to predict EQI. The meaning of all the feature labels is explained in the Supplementary Data S1–S4. The network representation in top panel is generated with Gephi 0.9.5^48^.

**Figure 5:**
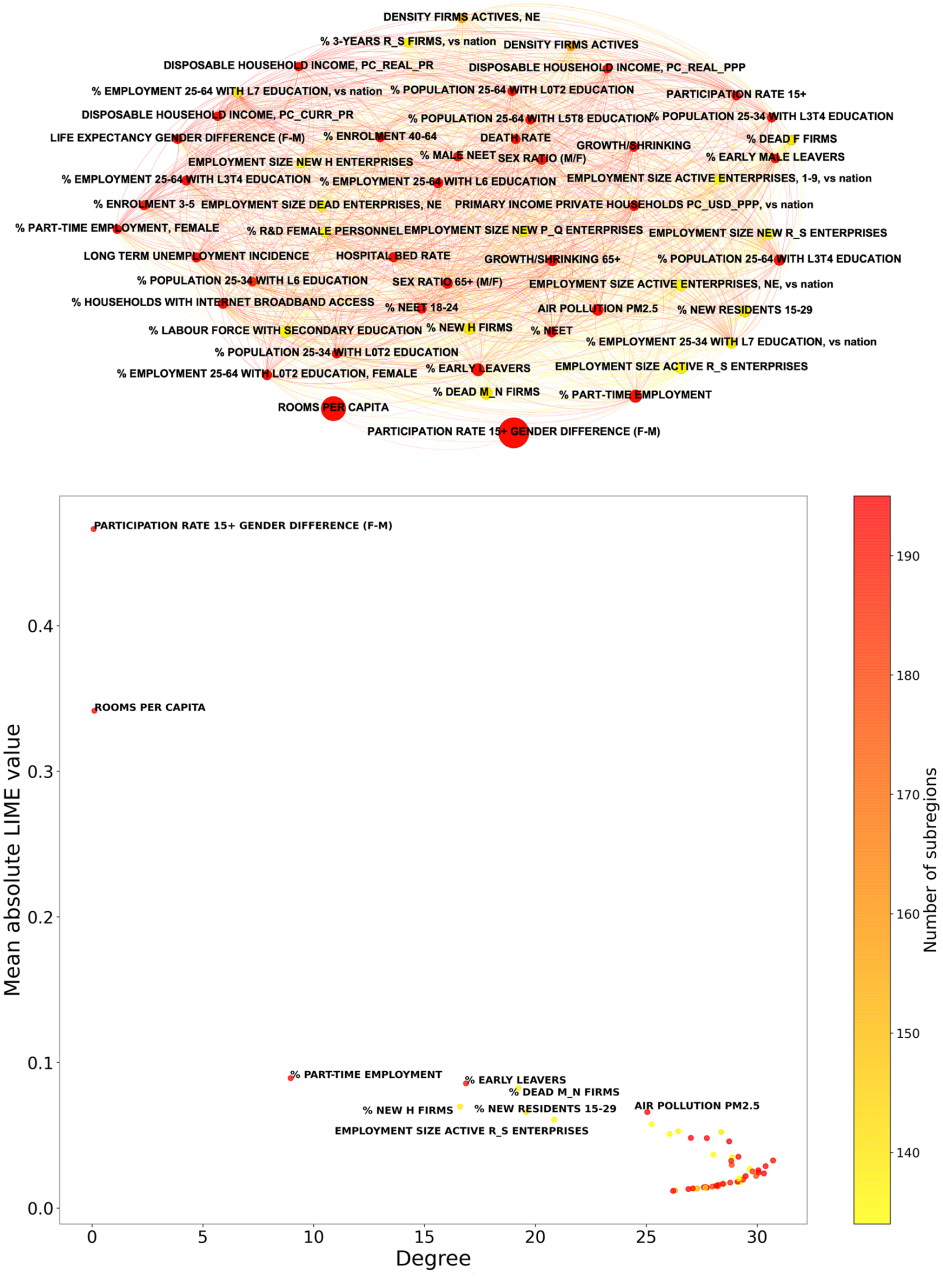
Top: competition network of the selected OECD indicators, determined from the importance rankings of their mean absolute LIME values for each subregion considered in the study. Bottom: scatter plot illustrating the relation between the mean absolute LIME value and the degree in the competition network, for indicator; labels are reported only for features whose mean absolute LIME value exceeds 0.06. In both panels, the color of points indicates the number of regions for which the considered indicator is available in the dataset. In the top panel, the size of each node increases with the mean absolute LIME value of the corresponding indicator. As in the case of SHAP values, the results confirm that both PARTICIPATION RATE 15+ GENDER DIFFERENCE (F-M) and ROOMS PER CAPITA are outstandingly relevant in their capability to predict EQI. The meaning of all the feature labels is explained in the Supplementary Data S1–S4. The network representation in top panel is generated with Gephi 0.9.5^48^.

In Figs. 4, 5, we show an illustration of the competition networks, along with scatter plots in which coordinates are the network degree and the mean absolute SHAP or LIME value, respectively, computed over the 195 regions. Points corresponding to indicators that are relevant in a stable way, for most of the regions, tend to be placed towards the upper left corner of the plot, in which a high mean absolute SHAP or LIME value is accompanied by a small network degree. Here, we find confirmation that both PARTICIPATION RATE 15+ GENDER DIFFERENCE (F-M) and ROOMS PER CAPITA are outstandingly relevant in their capability to predict EQI.

### Geographical analysis of SHAP and LIME values with a network of subregions

At this point, we investigate the possible relations between the SHAP and LIME values associated to a given indicator and the general features of the considered regions. To address this issue, we construct a network in which the 195 considered TL2 subregions represent nodes, while edges connecting different regions are meant to highlight their mutual similarity with respect to the set of 53 OECD indicators selected by Boruta. Such a network, whose construction process is detailed in the "Materials and methods" section, consists of 14015 links, whose strength is based on Pearson correlation between the selected indicator sets of the subregions they connect.

**Figure 6:**
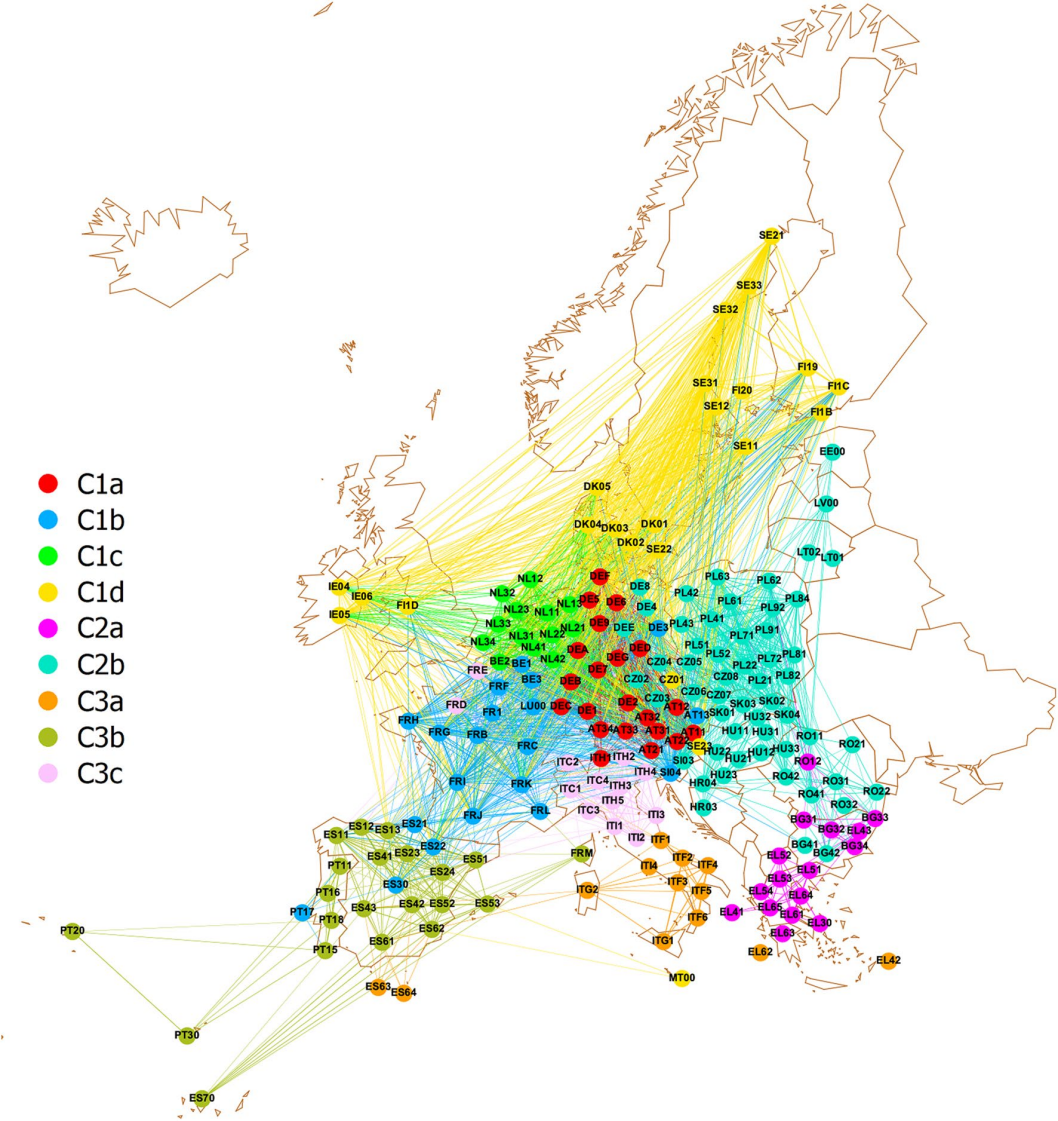
Complex network of regional similarities determined from the 53 OECD indicators selected by Boruta; node colors indicate the community membership of the 195 subregions. It is evident that, with the exception of few subregions, communities are very much influenced by geographical proximity. The map is generated with the “Map of Countries” and “GeoLayout” plugins of Gephi 0.9.5^48^.

We begin the analysis of region networks by investigating the possible relation between the EQI scores of territories and their similarity in terms of the 53 selected OECD indicators. The tendency in a network to connect nodes with similar attribute values is quantified by *assortativity* (see "Materials and methods" section for definition and characterization). In our case, we find that the region network is characterized by the statistically significant assortativity $0.258\pm 0.006$, associated with a *p*-value smaller than $10^{-9}$. Therefore, since connections among TL2 subregions are related in a relevant way to the results achieved in the EQI ranking, it is reasonable to expect that community detection tends to subdivide the network in groups of territories that are mostly homogeneous in their EQI outcomes. This framework allows to investigate the behavior of indicators that mostly impact on EQI prediction, within and across communities, to unveil possible patterns of similarities and differences in their SHAP and LIME values. Community detection in the subregion network provides the following partition:

- C1a: 21 regions in Austria (8), Germany (12), Italy (1);
- C1b: 20 regions in Austria (1), Belgium (2), Germany (1), Spain (3), France (10), Luxembourg (1), Portugal (1), Slovenia (1);
- C1c: 13 regions in Belgium (1), Netherlands (12);
- C1d: 23 regions in Czechia (1), Denmark (5), Finland (5), Ireland (3), Malta (1), Sweden (8);
- C2a: 16 regions in Bulgaria (4), Greece (11), Romania (1);
- C2b: 55 regions in Bulgaria (2), Czechia (7), Germany (3), Estonia (1), Croatia (2), Hungary (8), Lithuania (2), Latvia (1), Poland (17), Romania (7), Slovenia (1), Slovakia (4);
- C3a: 13 regions in Greece (2), Spain (2), Italy (9);
- C3b: 21 regions in Spain (14), France (1), Portugal (6);
- C3c: 13 regions in France (2), Italy (11).

The detailed composition of each community is represented in Fig. 6 and reported in Supplementary Data S5, while principles and intermediate results of the hierarchical procedure leading to the above communities are discussed in "Materials and methods".

The partition in communities represents the tool to analyze the relatedness between community membership and either the SHAP or the LIME values of the top-5 indicators in the respective mean absolute value rankings (see Fig. 3). The indicators involved in the SHAP geographical analysis are the following ones:

- PARTICIPATION RATE 15+ GENDER DIFFERENCE (F-M);
- ROOMS PER CAPITA;
- % EMPLOYMENT 25–34 WITH L7 EDUCATION, vs nation (employment rate for people in 25–34 year-old age range with master’s or equivalent level education—relative difference between subregional and national value);
- % LABOUR FORCE WITH SECONDARY EDUCATION (share of labour force with at least secondary education);
- % EARLY LEAVERS (rate of early leavers from education and training, in % of the total population aged 18 to 24).

On the other hand, the indicators involved in the LIME geographical analysis are the following ones:

- PARTICIPATION RATE 15+ GENDER DIFFERENCE (F-M);
- ROOMS PER CAPITA;
- % PART-TIME EMPLOYMENT (incidence of part-time employees over total employment);
- % EARLY LEAVERS;
- % DEAD M_N FIRMS (share of dead firms in the business population, in % of all firms, in the economic sectors of professional, scientific and technical activities, and administrative and support service activities).

**Table 1:** Resolution ratios *R* of the SHAP (LIME) distributions with respect to the partition of TL2 subregions in network communities, associated to the top-5 indicators in terms of mean absolute SHAP (LIME) values. Resolution ratios larger than 1, highlighted in boldface, indicate an average tendency of distributions pertaining to different communities to separate from each other. Missing entries correspond to the distributions of indicators that do not rank among top-5 in terms of either SHAP or LIME mean absolute values.

Indicator	$\varvec{R}$ (SHAP values)	$\varvec{R}$ (LIME values)
PARTICIPATION RATE 15+ GENDER DIFFERENCE (F-M)	$\varvec{2.157}$	$\varvec{1.817}$
ROOMS PER CAPITA	$\varvec{2.314}$	$\varvec{2.690}$
% EARLY LEAVERS	0.752	0.480
% EMPLOYMENT 25-34 WITH L7 EDUCATION, vs nation	0.226	—
% LABOUR FORCE WITH SECONDARY EDUCATION	$\varvec{1.151}$	—
% PART-TIME EMPLOYMENT	—	$\varvec{2.014}$
% DEAD M_N FIRMS	—	0.460

**Figure 7:**
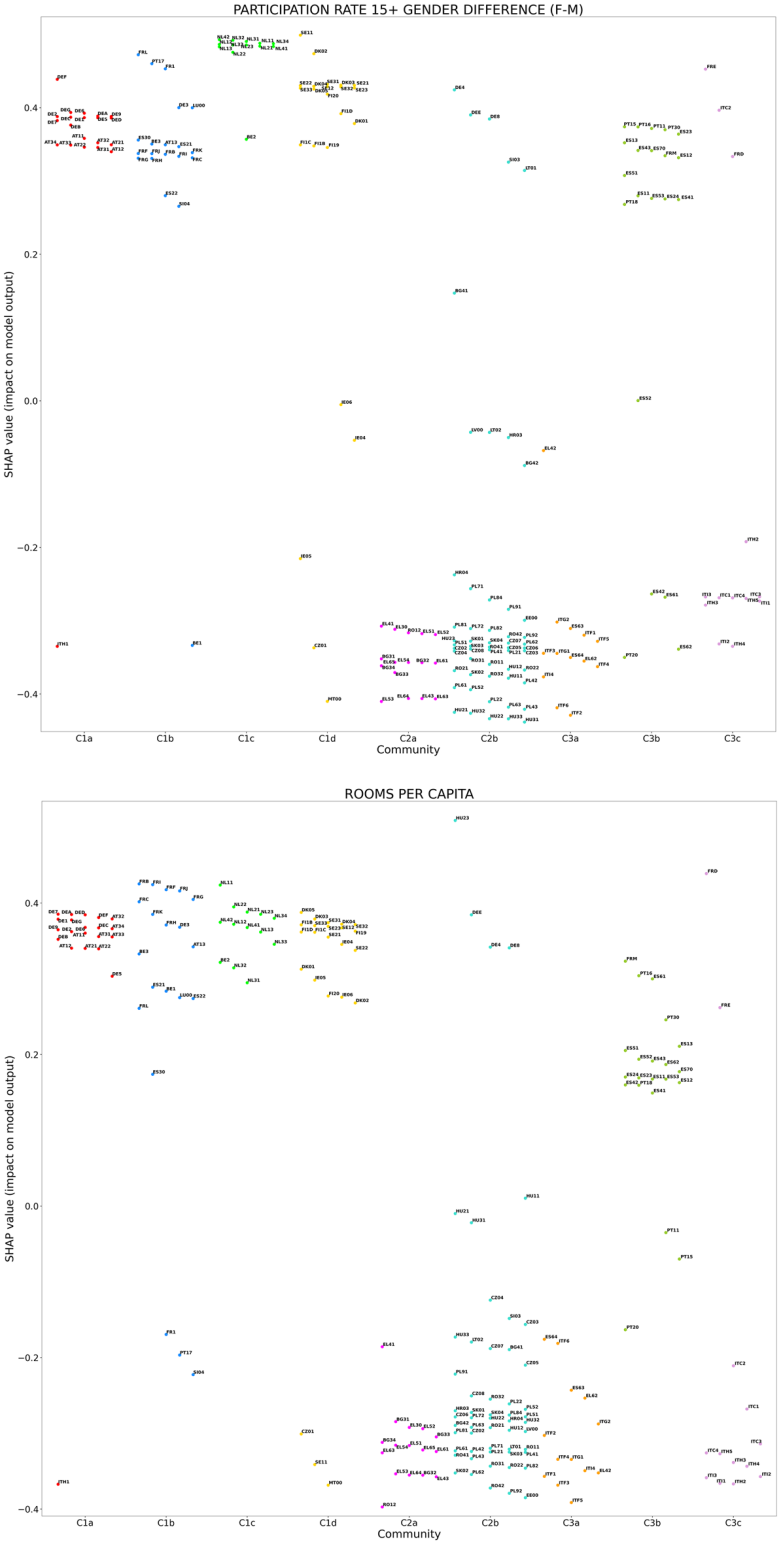
Distributions in the subregion network communities (reported on the horizontal axes) of the SHAP values related to the two indicators that show the highest impact, in terms of mean absolute SHAP value, on the prediction of EQI scores. In both cases, the distributions are characterized by $R>1$ with respect to the network partition, highlighting the tendency of indicators to have similar SHAP values in regions belonging to the same community and relevant differences across communities.

**Figure 8:**
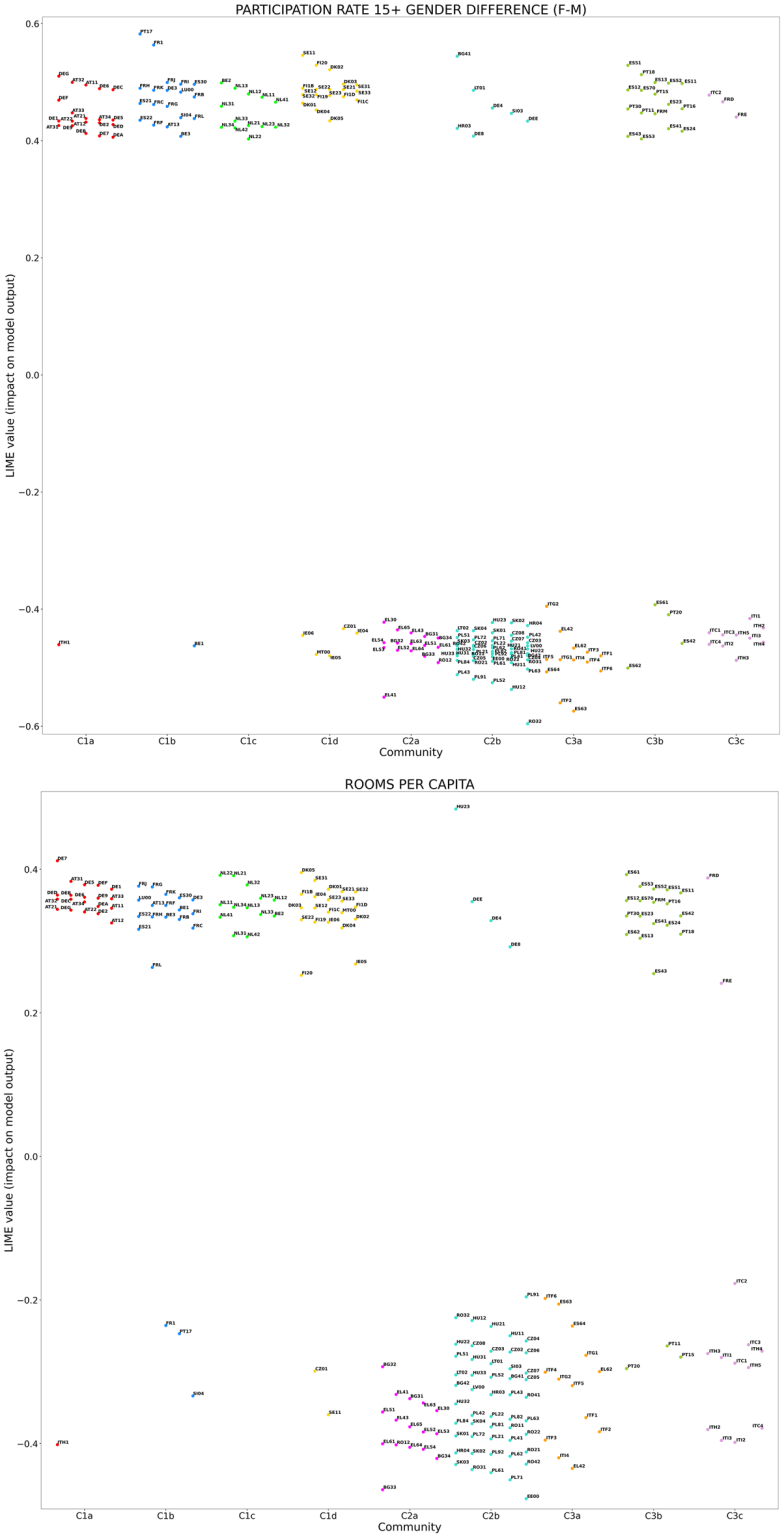
Distributions in the subregion network communities (reported on the horizontal axes) of the LIME values related to the two indicators that show the highest impact, in terms of mean absolute LIME value, on the prediction of EQI scores. In both cases, the distributions are characterized by $R>1$ with respect to the network partition, highlighting the tendency of indicators to have similar LIME values in regions belonging to the same community and relevant differences across communities.

In order to quantify the relatedness between SHAP/LIME values and community membership, we use the resolution ratio *R*, a quality factor introduced for a similar analysis in Ref.^38^, that increases as the distributions of a given index in different communities are more separated (see "Materials and methods" for definition and characterization). The resolution ratio is much larger than one when the community index distributions have a vanishing or limited overlap with each other, and much smaller than one when the overlap is practically full. In the intermediate case $R\simeq 1$ the separation between the mean values of neighboring community distributions is comparable to the typical variation of the index within each community. The value $R=1$ can thus be assumed as a threshold to distinguish cases ($R>1$) in which indicators show similar behaviors in regions belonging to the same community and relevant differences across communities, from cases ($R<1$) in which no such territorial pattern can be observed.

We determine the resolution ratio *R* for the SHAP and LIME value distributions of the indicators, listed above, that are ranked as top-5 in terms of SHAP and LIME mean absolute values, respectively. The results reported in Table 1 indicate that PARTICIPATION RATE 15+ GENDER DIFFERENCE (F-M) and ROOMS PER CAPITA correspond to distributions of both SHAP and LIME values that are well separated across communities, being characterized by $R>1$. These distributions are displayed in Figs. 7, 8. The analogous plots for the other indicators listed in Table 1 are reported in the Supplementary Information.

## Discussion

The various stages of our analysis highlight new findings on how territorial variables measuring development and individual well-being are related to the perception of government quality. In general, the study confirms that citizens’ evaluations on such an issue are often not directly related to the administrative action, but rather determined by the general objective conditions of a territory and people well-being.

The assortativity analysis on the subregion network suggests that regions for which the 53 selected indicators are highly-correlated tend to have comparable EQI values. This confirms the good predictive power of these indicators, independently emerging from XGBoost regression. The analysis of SHAP/LIME values related to such a prediction, corroborated by the indicator competition networks, points out the predominant role played by two indicators, namely PARTICIPATION RATE 15+ GENDER DIFFERENCE (F-M) and ROOMS PER CAPITA. It is also interesting that, as one can observe from Fig. 3, the impact on predictions of indicators related to the comparison with national values is limited (but not irrelevant), suggesting that the discrepancies between local conditions and those of the rest of a country play, on average, a minor role.

The emerging relevance of PARTICIPATION RATE 15+ GENDER DIFFERENCE (F-M) and ROOMS PER CAPITA in determining the perceived quality of government is highly noteworthy. The former indicator represents the difference between the labour participation rates, counting people that are either employed or actively searching for work, related to the female and male populations. The importance of such an indicator can be traced back to the fact that different relevant aspects contribute to its outcome, such as gender equity in society, which has one of its most convincing expressions in equal job opportunities, an open and vital labour market, welfare initiatives supporting maternity, and the availability of a larger number of income sources per household. In fact, despite the rising female involvement in the job market since the postwar period, the reversing education gaps and stricter gender equality legislation recently introduced in several OECD countries, residual gender gaps in labour participation seem to persist. Recent empirical evidence shows that there are two main reasons that might explain this phenomenon: the first one is the prevalent role of women in childcare and housework, along with its impact on work-life balance; the second one is represented by general attitudes toward gender roles in society and gender identity^49^. The combined effect of such strongly related phenomena on EQI through PARTICIPATION RATE 15+ GENDER DIFFERENCE (F-M) may be even amplified. Actually, women’s burden in childcare and housework may in turn affect employer evaluations, promotion standards and wages. On the other hand, progressive attitudes toward gender roles are positively associated with more equal gender outcomes in the labour market^50^. Thus, the impact of PARTICIPATION RATE 15+ GENDER DIFFERENCE (F-M) on perceived quality of government can be explained as the outcome of two interrelated effects: the efficiency of labour market policies that support women’s participation, such as adequate and affordable childcare services, and the ability of public institutions to hamper the transmission of gender norms and stereotypes across generations.

On the other hand, the relevance of ROOMS PER CAPITA reflects the widely recognized importance of the housing problem in the management of social pressure^51^. First of all, this quantity is an established proxy of living conditions. Eurostat, the statistical office of the European Commission^52^, uses overcrowding, defined as the lack of an adequate number of rooms, as one of the variables to characterize severe housing deprivation, among other factors such as leaking roof, rot in window frames or floor, lack of bathtub or shower unit and indoor flushing toilet for sole use of the household, and too dark dwellings. Scientific literature also shows that overcrowding is the dimension that contributes most to determine housing deprivation^53^. Therefore, increasing the number of rooms per capita may help individuals and households to overcome severe material deprivation; this in turn may act by improving satisfaction with the government in place. For instance, individuals may perceive that government interventions play a key role in helping them to gain freedom from material deprivation, thus increasing the quality of government perceived. A further aspect is the relevance of the number of rooms per capita in view of the health and socio-economic consequences of the recent COVID-19 pandemic. Actually, not only house overcrowding may amplify the spread of respiratory infections by preventing individuals from keeping the necessary distance, but it also affects quality of daily life and remote working, in case of lockdown or quarantine. By contrast, it is acknowledged that living in under-occupied dwellings is associated with lower levels of anxiety, and higher life satisfaction^54^. Thus, it can be argued that increasing the number of rooms per capita enhances the health and socio-economic conditions of individuals, which may induce them to express approval towards government lockdown interventions to fight the pandemic, with a consequent positive impact on the EQI score.

The picture is completed by the outcome of the geographical analysis, which highlights strength and weaknesses of local government policies throughout Europe. Actually, it is possible to observe from Figs. 7–8 that the values of PARTICIPATION RATE 15+ GENDER DIFFERENCE (F-M) and ROOMS PER CAPITA have a negative influence on the predicted EQI, as quantified by the negative SHAP/LIME values, for communities involving regions in south-eastern Europe, while they positively affect EQI elsewhere. It is noteworthy that the impact of such indicators in Spanish and Portuguese regions is entirely different, in positive, from that in countries such as Italy and Greece, which are frequently associated to them in terms of economic conditions.

A strength of this research work lies in its model-agnostic nature, that does not require *ad hoc* hypotheses on the functional dependence of the target variable (EQI, in this case) on the indicators used to predict it. The proposed approach enables to identify the objective factors that, in a variety of different territorial contexts, contribute to determine the perception of government quality by citizens, providing the methodological basis for the development of a quantitative tool for policy design. The analysis carried out in this paper can be useful from a policy perspective as it provides information on the variables over which one can operate in order to improve the citizens’ opinion on the quality of government. This study demonstrates that job gender equity and housing may be key determinants of EQI. We finally remark that the adopted methodology, which follows the paradigm of Artificial Intelligence for Social Good^55^ to investigate the relation between an index of social relevance and context variables, can be applied to different areas of the civic life, such as health, regarding in particular the incidence of specific diseases throughout the population^56,57^, and crime-related issues.

## Materials and methods

### Experimental design

Our analysis is aimed at investigating the main factors affecting the EQI, which quantifies the quality of government perceived by citizens of European Union regions on a subnational scale. The study, based on machine learning and complex network methods, consists of the following steps, summarized in Fig. 1:

- collecting (1) values of territorial indicators characterizing the OECD TL2 European Union regions, combining in a reasonable way data abundance and recentness, (2) values of the EQI for the same regions;
- removing irrelevant and redundant information from the set of collected territorial indicators trough a preprocessing pipeline and the Boruta feature selection algorithm;
- training a regression algorithm, capable of predicting the EQI values for each of the 195 subregions from the selected TL2 OECD indicators;
- implementing the XAI approach to identify through SHAP and LIME values the features that are most important to determine the EQI prediction;
- constructing competition networks, in which indicators represent nodes, to compare their impact, quantified by the mean absolute SHAP and LIME values, on the EQI prediction, taking into account the different data availability across regions;
- analysing the geographical variability in the relation between territorial indicators and EQI values by means of a complex network with nodes coinciding with the 195 considered TL2 subregions and connections among them based on similarity in terms of the selected OECD indicators; verifying the assortative behavior of such a subregion network with respect to the EQI values;
- using the unsupervised partition of the subregion network, obtained by a community detection algorithm, to divide regions in homogeneous groups in terms of territory indicators, in order to find relations between SHAP/LIME values and community membership.

In the following, we detail the implementation of the aforementioned process.

### Data collection and preprocessing

The EQI ranking for the year 2021 is retrieved from the dedicated website^1^. Details on the computation of this index for the considered year are reported in the working paper^58^ by the Quality of Government (QoG) Institute of University of Gothenburg. The EQI 2021 is available for 240 European Union regions, mostly corresponding to the OECD subregions at Territorial Level 2 (TL2). Exceptions are represented by Germany and Belgium, for which the EQI is reported with a finer geographical resolution, where some of the TL2 subregions are split in smaller parts. In these cases, we associate to a given TL2 subregion a weighted average of EQI values reported for its parts, with a weight proportional to population (details on the merged regions are provided in the Supplementary Information). In such a way, we associate an EQI value to 197 OECD subregions at TL2.

Territorial indicators are collected from the OECD Regional Statistics database^5^. We perform a preliminary availability check, in which we select only those indicators containing data for at least two thirds of the 197 TL2 regions. This process yields 1056 indicators, that we subdivide in four groups: 594 indicators in the Business and Economy (BE) group, 191 in Demography (D), 245 in Education and Labour (EL), and 26 in Territorial and People Well-being (TPW). After noticing that data are entirely missing in all groups for region CY00 (Cyprus), and in the TPW group for FRY (French overseas), we exclude these two regions from the analysis, which hereafter involves 195 regions.

Inside each of the aforementioned categories, we select the “intensive” indicators, namely the ones that do not directly depend on either the territorial extension or the population of a territory. This selection process leaves 292 indicators in the BE group, 58 in D, 183 in EL, and 17 in TPW. The remaining quantities are then subjected to a further selection inside each category, which eliminates those correlated to another indicator by a Pearson correlation larger than 0.9, which are assumed to carry redundant information. In cases of statistical redundancy, we retain the indicator with the wider data availability. This selection leaves 189 indicators in BE, 24 in D, 85 in EL, and 17 in TPW group.

At this point, we enrich the dataset with the “vs nation” indicators, representing the comparison of local values with the corresponding national counterparts. Specifically, each one of them is defined as the difference

5 $$\begin{aligned} I_{\text {vs nation}}(s) = \frac{I(s)-I(C)}{I(C)} \end{aligned}$$

between the value associated to a given indicator *I* and TL2 subregion *s* belonging to country *C*, and the national value *I*(*C*) of the same indicator, normalized to the latter. In principle, this operation could be done for all the indicators remaining in the dataset. However, we have to discard cases in which the national value is vanishing or unavailable, eventually obtaining 181 additional “vs nation” indicators in the BE group, 22 in D, 83 in EL, and 17 in TPW.

We conclude indicator preprocessing by observing that the TPW dataset contains three quantities that are determined by perception rather than objective data, namely SELF-EVALUATION OF LIFE SATISFACTION, PERCEPTION OF CORRUPTION, PERCEIVED SOCIAL NETWORK SUPPORT. In order to minimize the conceptual overlap between features and the EQI index and avoid data leakage issues, we discard these three indicators, along with the corresponding ones compared with the national values.

### Feature selection

The data processing described in the previous subsection leads to an overall set of 612 indicators, whose lists, one for each category (BE, D, EL, TPW), are reported in Supplementary Data S1–S4. Before training the regression algorithms, we discard irrelevant features by applying the Boruta framework. Boruta is a feature selection wrapper method^6^ that enables to reduce at the same time noise and data redundancy, by selecting a set of uncorrelated features that improves in a statistically significant way the performance of a given machine learning algorithm. In our case, we make the standard choice of basing Boruta on a supervised learning Random Forest (RF) algorithm, which makes the results of feature selection independent of those obtained by XAI, where a different procedure is used. Boruta associates to the original feature set an equal number of *shadow* features, obtained by randomly shuffling the original set of indicator values. Such an extended dataset is then used to train a RF algorithm, which evaluates the importance of all features, including original and shadow ones, in predicting a given quantity. In this way, the most important features are identified, after independent random shuffling runs, as the ones that are more relevant, in a statistically significant way, than their shadow counterparts. This implies that the Boruta feature selection process is independent of any arbitrary importance threshold.

Regardless of the machine learning algorithm on which it is based, Boruta is not able to direclty handle unavailable feature values. Therefore, only in the context of Boruta feature selection, we choose to fill all the voids of a given indicator with the average of the available values. Boruta is run with estimator $=$ estimator_forest, n_estimators $=$ ‘auto’, max_iter $= 100$; the RF algorithm is run with parameters n_jobs $= -1$, max_depth $= 5$; the remaining parameters are set to default values. Out of 612 features, Boruta identifies 53 relevant ones, which are forwarded to XGBoost regression.

### XGBoost algorithm

The XGBoost algorithm, used in the regression task to predict EQI from the 53 indicators selected by Boruta, was chosen in order to automatically manage unavailable values, without artificial filling. This machine learning approach is based on an ensemble of decision trees, weak prediction models that are iteratively trained through a gradient boosting pipeline, in which new decision trees specialize in addressing the critical points of the previous ones. In this process, the XGBoost algorithm is able to tackle the problem of missing values by sparsity-aware split finding, which exploits the data sparsity pattern in a unified way, learning the best direction to take when the feature needed for the split is missing^59^. We determined the best performance of regression in the leave-one-out mode by exploring combinations of the following XGBoost parameters:

- num_parallel_tree $$\in \{50,100,250\}$$,
- max_depth $$\in \{2,5,7\}$$,
- n_jobs $$\in \{1,5,10,100\}$$,

with importance_type set to “gain” mode and squared error as the default objective function. The optimal configuration is found for num_parallel_tree$=100$, max_depth$=2$, independently of n_jobs. The regression outcomes corresponding to these values are reported in the "Results" section, and illustrated in Fig. 2. The variability with respect to the best performance quantifiers in the explored parameter space is small, with standard deviations amounting to $1.7\%$ of the optimal $r^2$, $0.9\%$ of the optimal *P*, $4.1\%$ of the optimal *MAE*, and $6.4\%$ of the optimal *RMSE*; notice that the results do not depend on the n_jobs parameter.

### Explainable Artificial Intelligence

We describe here the principles and the operation of the XAI algorithms employed in this research, namely SHAP and LIME.

SHAP (SHapley Additive exPlanation) is an algorithm based on the concept of the Shapley values derived from the cooperative game theory^60,61^. It is a local model-agnostic post-hoc explainer, hence it can learn local interpretable linear models for the samples, ignoring the specific regressors (or classifiers) to explore the contribution of each feature on the prediction of each sample. The Shapley value for a feature *j* is assessed as the difference between the prediction of the model output by including that feature and the prediction of the model without that feature, considering all possible feature subsets with and without *j*. In order to assess the Shapley values, the model should be retrained on all feature subsets *F*, included in the total set *S* of features ($F \subseteq S$). For a generic model *f*, if $f_{x}(F)$ denotes the prediction for the instance *x* given the subset *F* without the *j*-th feature, and $f_{x}(F \cup {j})$ denotes the prediction resulting by adding the *j*-th feature to the subset *F*, the marginal contribution of adding the *j*-th feature value to *F* can be computed as the difference $f_{x}(F \cup {j})-f_{x}(F)$. The Shapley value, measuring the impact of the *j*-th feature on the prediction for *x*, is then assessed by considering all possible subsets of feature values:

6 $$\begin{aligned} SHAP_j(x) = \sum _{F\subseteq S - \{j\}} \frac{|F|!(|S|-|F|-1)!}{|S|!} [f_{x}(F \cup {j})-f_{x}(F)] , \end{aligned}$$

where |*F*|! represents the number of permutations of features positioned before the *j*-th feature, $(|S|-|F|-1)!$ represents the number of permutations of feature values that appear after the *j*-th feature value and |*S*|! is the total number of permutations^60^.

LIME (Local Interpretable Model-agnostic Explanations) represents a different model-agnostic explanation method, whose goal is to associate to a regressor (or a classifier) *f* an interpretable model *g*, found through an optimization process^62^. The input of *g* is represented by the *simplified input features* $z'\in Z$, namely binary vectors in which a component is 1 if the corresponding feature is made available to *f*, and 0 otherwise. The procedure is based on finding, for a given input instance *x*, the parameters of *g* that minimize the objective function

7 $$\begin{aligned} \sum _{z'\in Z} \pi _x(z') \left( g(z') - f_x(z') \right) ^2 + \Omega (g). \end{aligned}$$

In the above equation, $\pi _x(z')$ is a weight function which adds a higher penalty for vectors $z'$ that are farther from the simplified feature vector $x'$, whose components are either 1 or 0 according to whether the corresponding feature is available or not in the input *x*. The function $\Omega (g)$, instead, quantifies the complexity of the model (e.g., the number of non-zero weights in a linear model, or the tree depth in decision trees), that is usually fixed *a priori*. If *g* is a linear model, the weights of features in the prediction for input *x* are identified as the corresponding linear coefficients. In this work, we employed a linear model through the function LimeTabularExplainer of the “lime” package for Python^63^, setting the regression mode and using default settings for the other parameters; the function uses, in default mode, an exponential weight in the Euclidean distance between vectors $x'$ and $z'$, whose width is set to 0.75 times the square root of the total number of features.

### Indicator competition network construction

The indicator competition network is based on comparing their rankings in the 195 SHAP or LIME lists associated to the EQI prediction for each considered region. For definiteness, we focus on the case of SHAP, the procedure followed for LIME being identical. For each pair of indicators *i* and *j*, corresponding to nodes in the network, we can associate the values $SHAP_i(\alpha )$ and $SHAP_j(\alpha )$, with $\alpha \in \{1,2,\dots ,195\}$ labelling the region. Then, we construct a 195-element vector $V_{ij}$, whose $\alpha$-th entry is 0 if $SHAP_i(\alpha )>SHAP_j(\alpha )$, and 1 in the complementary case. The variety of entries in $V_{ij}$ is quantified by its Shannon entropy, which constitutes the weight of the edge connecting nodes *i* and *j*. Therefore, if *i* is more relevant than *j* in the EQI prediction for all regions, then $V_{ij}$ will contain only zeros, and its entropy will be vanishing. The same occurs in the opposite case, in which *j* is always more relevant. In these two extreme cases, there is no link between *i* and *j* (i.e., they do not compete with each other). Such a network construction process provides 1357 links for SHAP and 1265 links for LIME.

### Region network construction

Using the 53 indicators selected by Boruta, a region network is constructed, whose nodes correspond to the 195 considered TL2 regions. In principle, in this network, each pair of regions can be connected, with edge weight provided by the Pearson correlation between the two vectors containing their territorial indicators. However, we choose to discard a given edge in case the null hypothesis of uncorrelated sets of indicators cannot be rejected at a significance level of 5% (i.e., when $p>0.05$, according to the test based on comparing the sample Pearson correlation with the exact distribution of the correlation values between two random vectors, independent and normally distributed). Therefore, the region network is not complete, as reported in the "Results" section, and edges can have both positive and negative weights. Notice that each indicator distribution is normalized in a way that 0 and 1 correspond to the minimum and maximum values, respectively.

### Assortativity

Since we generally deal with weighted network, we consider the generalized definition of assortativity with respect to an attribute *X*^45^, which takes the value $x_i$ in correspondence of the *i*-th node:

8 $$\begin{aligned} r_w = \frac{ \sum _{ij} \left( w_{ij} - \frac{s_i s_j}{W} \right) x_i x_j }{ \sum _{ij} \left( s_i \delta _{ij} - \frac{s_i s_j}{W} \right) x_i x_j } . \end{aligned}$$

In the above definition, $w_{ij}$ is the weight of the edge (*i*, *j*), $s_i=\sum _j w_{ij}$ is the strength of node *i*, and $W=\sum _{ij} w_{ij}$. The expression of $r_w$ remains meaningful only in the case of positive weights. Therefore, before evaluating assortativity, we associate to the considered network, which can contain negative-weight edges, an auxiliary subnetwork where only positive-weight edges are retained.

The values of assortativity range from $-1$ (maximally antiassortative network) to $+1$ (maximally assortative). A network consisting of two same-size and fully connected components, with no connection between them and characterized by two different values of an attribute, is maximally assortative. A network consisting of two subsets with the same cardinality, each characterized by a specific attribute value, with no internal connection but with each node only connected to nodes of the other subset, is maximally antiassortative.

The definition (8) is formally equivalent to the weighted Pearson correlation between two vectors of length 2*m*, with *m* the number of edges, whose entries coincide respectively with the attributes $x_i$ and $x_j$ of the nodes at the ends of each edge (*i*, *j*); the contribution of a given pair $(x_i,x_j)$ to the overall correlation is determined by the weight $w_{ij}$ of the corresponding edge. If $r_W=0$, there is no relevant linear correlation between the attribute vectors of nodes connected by edges. The interpretation of assortativity in terms of a weighted Pearson correlation allows to associate to $r_w$ the standard error

9 $$\begin{aligned} S_w = \sqrt{ \frac{1-r_w^2}{2m-2} } , \end{aligned}$$

that is evaluated, along with the related *p*-value, based on the Student *t*-distribution hypothesis^64^. The assortativity and the associated standard errors and *p*-values are computed through an algorithm implemented in the “weights” R library^65^.

### Community detection

Community detection is performed using the Spin Glass algorithm^66,67^, with the implementation mode that enables to consider links with negative weights. The resolution $\gamma$ is treated as a free parameter, varied in the interval [0.8, 1] with step width 0.05. The other parameters of the algorithm are fixed to default values: the upper limit for the number of communities is equal to the number of network nodes (195 in the region networks); the $\lambda$ argument, that specifies the balance between the importance of present and missing negatively-weighted edges within a community, is set to 0.01; the null model is a random graph with the same vertex degrees as the input graph; the cooling factor for the simulated annealing process is set to 0.5.

For a given set of parameters, we perform $K=100$ runs of the algorithm, each one with a different seed of the pseudorandom number generator. The partition in communities is then chosen by majority voting. However, in order to evaluate the stability of the detected partition in communities, we do not just count the frequency of the most frequently recurring partition. Instead, we introduce a new stability criterion, that takes into account the similarity between different partitions $\{p_j\}_{(j=1,..,K)}$, based on the average Normalized Mutual Information

10 $$\begin{aligned} \left\langle NMI \right\rangle = \frac{2}{K(K-1)} \sum _{a=1}^{K-1} \sum _{b=a+1}^K NMI(p_a,p_b) , \end{aligned}$$

where $NMI(p_a,p_b)$ is the Normalized Mutual Information between a given pair of partitions, and $K(K-1)/2$ is the number of distinct pairs. The majority partition over $K=100$ runs is approved under the condition $\langle NMI \rangle \ge 0.90$, related to the general stability of the community detection. Moreover, the majority partition must satisfy the following further requirements:

1. it must not be trivial (i.e., consisting of a single community, coinciding with the whole network);
2. it must not be too fragmented, containing communities whose cardinality is less than 5% of the cardinality of the whole network (i.e., less than 10 nodes).

If the results obtained for 100 runs, at different values of the resolution $\gamma$, satisfy the above conditions, we choose the output with larger $\langle NMI \rangle$, and the majority partition corresponding to this choice is identified as the result of community detection.

Community detection is performed following a hierarchical procedure: at the first step, the entire network is partitioned according to the above method. Then a second community detection algorithm is performed on each of the first-level communities, and so on to the next levels. A branch of network partitions interrupts if at least one of conditions 1 and 2 is violated; this does not affect the progress of hierarchical partition for other branches.

Considering that the hierarchical community detection on the subregion network does not find partitions beyond the second step, we label each community with a capital “C”, followed by a number and a lower-case letter; communities labelled with the same number derive from the same community found at the first hierarchical level. For example, communities C1a and C1b are obtained at the second hierarchical level by subdividing the same community “1” found at the first hierarchical level. Communities C2a and C3a, instead, do not derive from the same first-level community, and the coincidence of their lower-case letters is accidental.

Hierarchical community detection proceeds as follows:

1. the whole network is partitioned in the 3 communities C1 (77 nodes), C2 (71 nodes), and C3 (47 nodes), for $\gamma =1.00$, with 59% agreement and $\langle NMI \rangle = 0.923$;
2. community C1 is partitioned in the 4 communities C1a (21 nodes), C1b (20 nodes), C1c (13 nodes) and C1d (23 nodes), for $\gamma = 1.05$, with 93% agreement and $\langle NMI \rangle =0.973$; community C2 is partitioned in the 2 communities C2a (16 nodes) and C2b (55 nodes), for $\gamma =0.90$, with 98% agreement and $\langle NMI\rangle =0.995$; community C3 is partitioned in the 3 communities C3a (13 nodes), C3b (21 nodes) and C3c (13 nodes), for $\gamma =1.00$ and $\langle NMI\rangle =0.999$;
3. no third-level division of any community found at the second level satisfies both acceptance criteria.

### Resolution ratio

The resolution ratio *R* can be used to quantitatively characterize the separation between ranked index distributions pertaining to different communities. The quantity is defined in terms of mean and variance of the community distributions and of the overall distribution. The definition of the resolution ratio *R* is based on the relation between the values $\{x_i\}$ assigned to each element $i=1,\dots ,N$ of a given set and the partition of that set in *K* disjoint groups of cardinality $n_c$, with $c=1,\dots ,K$. Given the values and the partition, one can evaluate the overall mean $\mu$ and variance $\sigma ^2$ of the whole set $\{x_i\}$, as well as the mean $\mu _c$ and variance $\sigma ^2_c$ for each group *c*. The overall variance $\sigma ^2$ can be separated in two positive contributions

11 $$\begin{aligned} \sigma ^2=\sigma ^2_{\textrm{int}} + \sigma ^2_{\textrm{ext}}, \qquad \text {with} \quad \sigma ^2_{\textrm{int}} = \sum _{c=1}^K \frac{n_c}{N} \sigma ^2_c, \quad \sigma ^2_{\textrm{ext}} = \sum _{c=1}^K \frac{n_c}{N} (\mu _c-\mu )^2 . \end{aligned}$$

Since $\sigma ^2_{\textrm{int}}$ coincides with a weighted average of group variances, while $\sigma ^2_{\textrm{ext}}$ represents the contribution determined by the discrepancy between the group means and the overall mean, a good indicator of separation of group distributions is given by $R=\sigma ^2_{\textrm{ext}}/\sigma ^2_{\textrm{int}}$.

## Supplementary Information

Supplementary Information 1.

Supplementary Information 2.

Supplementary Information 3.

Supplementary Information 4.

Supplementary Information 5.

Supplementary Information 6.

## Data Availability

The data that support the findings of this study are either publicly available on databases cited in the bibliography, or reported in Supplementary Data files. The source code and the data required to run it are available at the following URL: 10.5281/zenodo.7504274.
