# Supplementary material for: Detecting the socio-economic drivers of confidence in government with eXplainable Artificial Intelligence
Source: Sci Rep. 2023 Jan 16;13:839. doi: 10.1038/s41598-023-28020-5 (PMC9841965; doi:10.1038/s41598-023-28020-5)
Supplement: Supplementary file 1 — Supplementary Information 1. [file 41598_2023_28020_MOESM1_ESM.pdf]

SUPPLEMENTARY INFORMATION
Detecting the socio-economic drivers of confidence in government with eXplainable Artificial Intelligence

Loredana Bellantuono[1,2,*], Flaviana Palmisano[3], Nicola Amoroso[4,2], Alfonso Monaco[5,2], Vitorocco Peragine[6,†], and Roberto Bellotti[5,2,†]

[1]Dipartimento di Biomedicina Traslazionale e Neuroscienze (DiBraiN), Università degli Studi di Bari Aldo Moro, 70124, Bari, Italy
[2]Istituto Nazionale di Fisica Nucleare, Sezione di Bari, 70125, Bari, Italy
[3]Department of Economics and Law, Sapienza University of Rome, 00161, Roma, Italy
[4]Dipartimento di Farmacia-Scienze del Farmaco, Università degli Studi di Bari Aldo Moro, 70125, Bari, Italy
[5]Dipartimento Interateneo di Fisica, Università degli Studi di Bari Aldo Moro, 70126, Bari, Italy
[6]Dipartimento di Economia e Finanza, Università degli Studi di Bari Aldo Moro, 70124, Bari, Italy
[*]loredana.bellantuono@ba.infn.it
[†]These authors contributed equally to this work.

ABSTRACT

In this Supplementary Information document, we report complementary and technical information that corroborates the results presented in the main text.

EQI value for TL2 subregions

The 2021 EQI value is reported for European Union regions that mostly coincide with OECD subregions at the Territorial Level 2 (TL2). An exception is represented by a group of regions in Germany and Belgium, which are split in smaller parts. The EQI of the TL2 subregions in the aforementioned countries is evaluated by a weighted average of the EQI values of their finer subdivisions, with weights provided by the population in each of these parts.

Specifically, the EQI is reported for:

- BE21, BE22, BE23, BE24, BE25, belonging to the TL2 subregion BE2 (Belgium);
- BE31, BE32, BE33, BE34, BE35, belonging to the TL2 subregion BE3 (Belgium);
- DE11, DE12, DE13, DE14, belonging to the TL2 subregion DE1 (Germany);
- DE21, DE22, DE23, DE24, DE25, DE26, DE27, belonging to the TL2 subregion DE2 (Germany);
- DE71, DE72, DE73, belonging to the TL2 subregion DE7 (Germany);
- DE91, DE92, DE93, DE94, belonging to the TL2 subregion DE9 (Germany);
- DEA1, DEA2, DEA3, DEA4, DEA5, belonging to the TL2 subregion DEA (Germany);
- DEB1, DEB2, DEB3, belonging to the TL2 subregion DEB (Germany);
- DED2, DED4, DED5, belonging to the TL2 subregion DED (Germany).

SHAP value distributions in TPW communities for additional indicators

For the sake of completeness, we report in Fig. S1 the SHAP value distributions in region network communities for the indicators ranked from third to fifth in terms of mean absolute SHAP, and in Fig. S2 the LIME value distributions in region network communities for the indicators ranked from third to fifth in terms of mean absolute LIME. The corresponding values of resolution ratio R are reported in Table 1 of the main text.

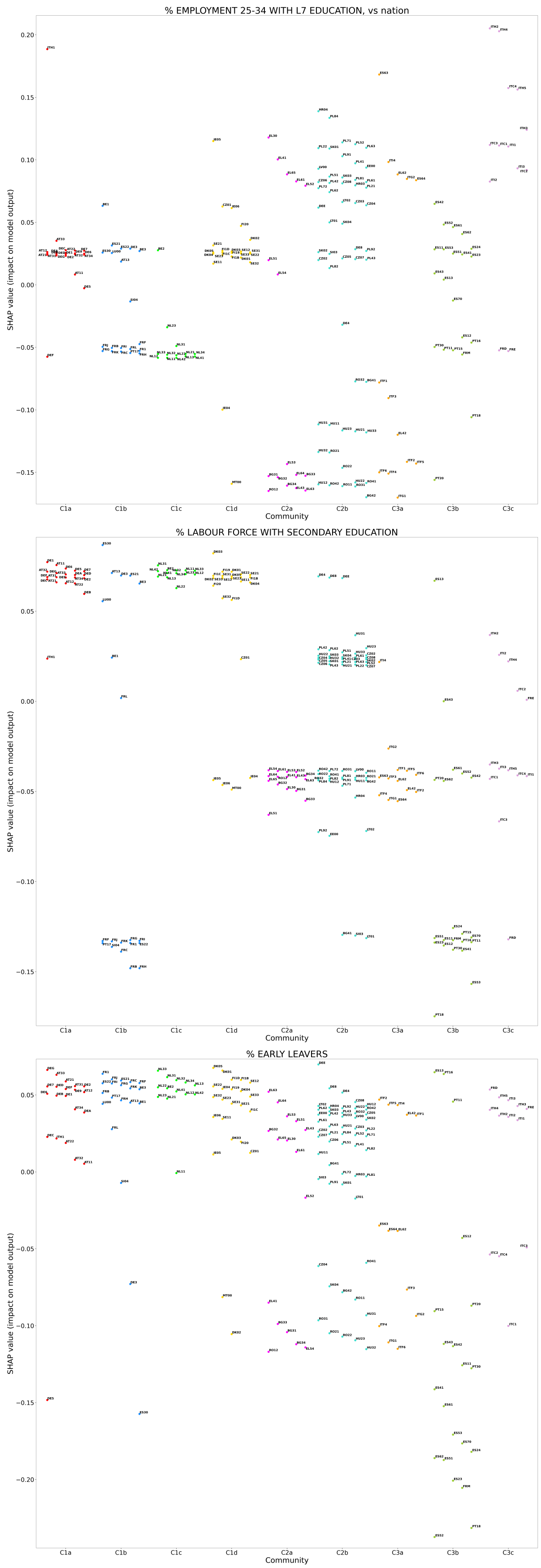

Figure S1. Distributions in the subregion network communities (reported on the horizontal axes) of the SHAP values related to the indicators ranked from third to fifth in terms of mean absolute SHAP.

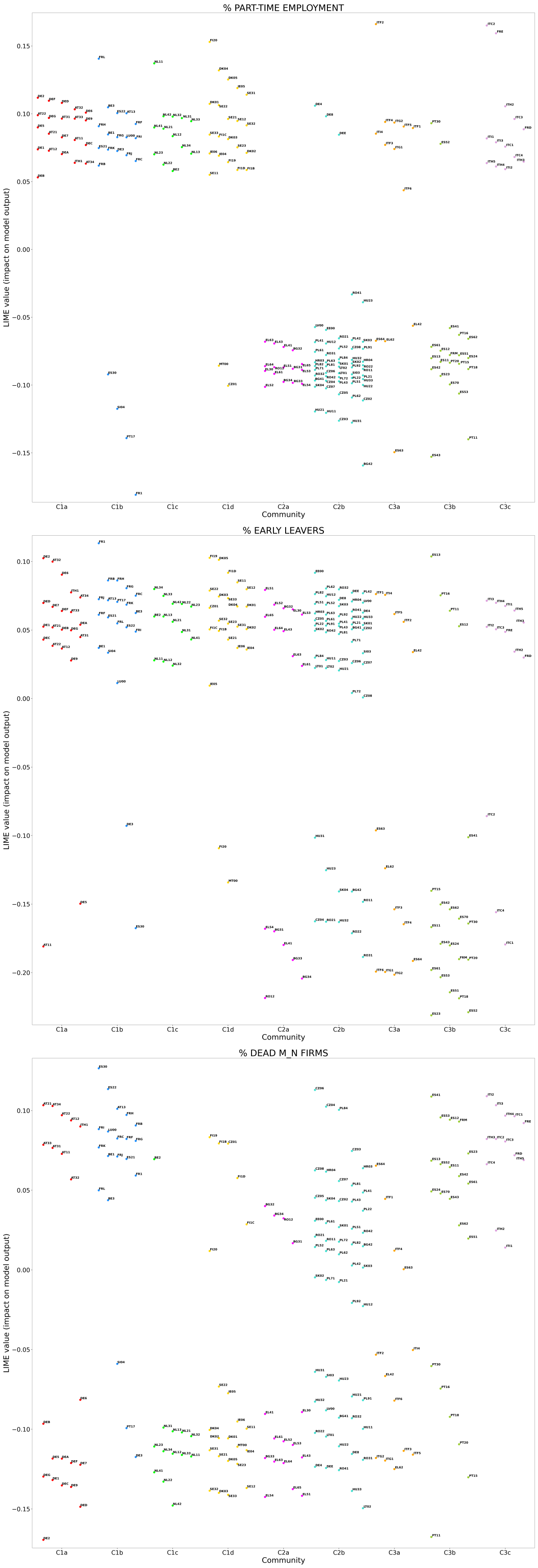

Figure S2. Distributions in the subregion network communities (reported on the horizontal axes) of the LIME values related to the indicators ranked from third to fifth in terms of mean absolute LIME.

Supplementary Data files

We report below a list of the Supplementary Data files attached to this submission:

- **Supplementary Data S1** (file Data_S1.xlsx): List of the subregional indicators belonging to the BE category, including indicators referred to the national values, provided in input to Boruta; selected indicators are highlighted in cyan.
- **Supplementary Data S2** (file Data_S2.xlsx): List of the subregional indicators belonging to the D category, including indicators referred to the national values, provided in input to Boruta selected indicators are highlighted in cyan.
- **Supplementary Data S3** (file Data_S3.xlsx): List of the subregional indicators belonging to the EL category, including indicators referred to the national values, provided in input to Boruta selected indicators are highlighted in cyan.
- **Supplementary Data S4** (file Data_S4.xlsx): List of the subregional indicators belonging to the TPW category, including indicators referred to the national values, provided in input to Boruta selected indicators are highlighted in cyan.
- **Supplementary Data S5** (file Data_S5.xlsx): List of the 195 subregions considered in the analysis, with their TL2 code, their full name and their community membership in the subregion network.
